# Risk of Endometrial Polyps, Hyperplasia, Carcinoma, and Uterine Cancer After Tamoxifen Treatment in Premenopausal Women With Breast Cancer

**DOI:** 10.1001/jamanetworkopen.2022.43951

**Published:** 2022-11-28

**Authors:** Ki-Jin Ryu, Min Sun Kim, Ji Yoon Lee, Seunghyun Nam, Hye Gyeong Jeong, Tak Kim, Hyuntae Park

**Affiliations:** 1 Department of Obstetrics and Gynecology, Korea University Anam Hospital, Korea University College of Medicine, Seongbuk-gu, Seoul, Republic of Korea; 2 Department of Biostatistics, Korea University Anam Hospital, Korea University College of Medicine, Seongbuk-gu, Seoul, Republic of Korea

## Abstract

**Importance:**

The association of tamoxifen use with the risk of uterine diseases, such as endometrial cancer, in premenopausal women with breast cancer remains controversial. However, many studies have reported an increased risk of uterine disease among postmenopausal tamoxifen users.

**Objective:**

To investigate the association of tamoxifen use with the risk of endometrial cancer and other uterine diseases in premenopausal women with breast cancer.

**Design, Setting, and Participants:**

A nationwide, population-based, retrospective longitudinal cohort study with an 18-year study period was conducted using data obtained from the Korean National Health Insurance Service. Participants included premenopausal women aged 20 to 50 years with breast cancer diagnoses between January 2003 and December 2018. Data were analyzed from April to December 2021.

**Exposures:**

Tamoxifen treatment.

**Main Outcomes and Measures:**

The incidence of uterine diseases, including endometrial cancer, hyperplasia, polyps, and other uterine cancers, was identified in the study cohort using insurance claim codes. The incidence of uterine diseases per 1000 person-years was compared between women receiving tamoxifen and those not treated with adjuvant hormone therapy. Multivariable Cox proportional hazard regression analysis was performed to determine the risk of each uterine disease.

**Results:**

Among 78 320 female participants with a mean (SD) age of 42.1 (6.1) years, 34 637 (44.2%) were categorized into the tamoxifen group and 43 683 (55.8%) were categorized into the control group. Among tamoxifen users, during the mean (SD) follow-up duration of 6.13 (4.15) years, the incidence of newly diagnosed endometrial polyps was 20.13 cases per 1000 person-years, that of endometrial hyperplasia was 13.49 cases per 1000 person-years, that of endometrial cancer was 2.01 cases per 1000 person-years, and that of other uterine cancers was 0.45 cases per 1000 person-years. The risk of endometrial cancer was higher in the tamoxifen group than in the control group (hazard ratio, 3.77; 95% CI, 3.04-4.66) after adjusting for age, body mass index, history of diabetes, hypertension, dyslipidemia, polycystic ovary syndrome, gonadotropin-releasing hormone agonist treatment, and trastuzumab treatment.

**Conclusions and Relevance:**

In this longitudinal cohort study, premenopausal Korean women with breast cancer who received tamoxifen as adjuvant hormone therapy had a significantly increased risk of endometrial hyperplasia, polyps, carcinoma, and other uterine cancers compared with those who were not treated with adjuvant hormone therapy. These findings suggest that clinicians should consider the risk of uterine disease among tamoxifen users, including premenopausal women.

## Key Points

**Question:**

Is the use of tamoxifen as an adjuvant hormone therapy for breast cancer associated with increased risk of uterine diseases among premenopausal Korean women?

**Findings:**

This cohort study of 78 320 participants revealed that tamoxifen use in premenopausal women with breast cancer was independently associated with an increased risk of endometrial polyps, hyperplasia, carcinoma, and other uterine cancers.

**Meaning:**

These findings suggest that awareness about the absolute risks of uterine diseases with long-term follow-up is essential for the daily management of premenopausal breast cancer survivors receiving tamoxifen and that the risk of uterine diseases in tamoxifen users, specifically in premenopausal women, should be considered.

## Teaser

This cohort study evaluates the risk of endometrial polyps, hyperplasia, carcinoma, and uterine cancer after tamoxifen treatment among premenopausal Korean women with breast cancer.

## Introduction

Tamoxifen was the first selective estrogen receptor modulator approved by the US Food and Drug Administration as an adjuvant hormone therapy for women with breast cancer. Worldwide, breast cancer is the most common cancer in women.^1,2^ The National Comprehensive Cancer Network Guidelines suggest the use of tamoxifen for 5 years as adjuvant therapy for premenopausal women with estrogen receptor–positive invasive breast cancer or ductal carcinoma in situ.^3^ Women who remain premenopausal after the first course can consider tamoxifen for another 5 years, whereas postmenopausal women can consider tamoxifen for 5 years or switch to an aromatase inhibitor.^3^ Although tamoxifen can block the effects of estrogen in the breast tissue, it acts like estrogen in the uterus. It is assumed to have tumor-promoting activity, which is associated with serious adverse effects.^4^ Indeed, tamoxifen use has been reported to be associated with various uterine pathologies, such as endometrial polyps, hyperplasia, cancer, and other uterine cancers, in postmenopausal women.^4^ A meta-analysis^5^ of 55 randomized trials showed that the incidence of endometrial cancer increased, and the risk of mortality owing to endometrial cancer increased after the administration of adjuvant tamoxifen therapy in postmenopausal women with early breast cancer.

Although premenopausal women with breast cancer are usually treated with tamoxifen as first-line adjuvant hormone therapy,^3^ it remains unclear whether the use of tamoxifen in premenopausal women is associated with an increased risk of several uterine diseases, including endometrial cancer. Although many reports have described an increased risk of endometrial cancer in tamoxifen users, the risk group has typically been confined to postmenopausal women with breast cancer.^6^ Few retrospective or case-control studies have assessed endometrial pathology in premenopausal women treated with tamoxifen.^7,8,9,10,11^ Existing guidelines do not warn of the potential risk of gynecologic diseases with tamoxifen use, nor do they recommend routine screening for uterine diseases in premenopausal women receiving tamoxifen.^12,13^ Furthermore, Asian women are known to have an earlier peak age of occurrence of breast cancer than Western women, suggesting that the proportion of premenopausal women among tamoxifen users is higher in Asian women.^14^ However, extrapolation of the observations in postmenopausal Western women to relatively young premenopausal Asian women is debatable. Therefore, the objective of this cohort study was to use Korean nationwide cohort data to examine the risks of uterine pathologies, including endometrial hyperplasia, polyps, carcinoma, and other uterine cancers, in premenopausal Korean women with breast cancer who had been treated with tamoxifen.

## Methods

### Data Source

We used data obtained from the Korean National Health Insurance Service (NHIS), a single medical insurer in the Republic of Korea. This national insurer is operated by the Ministry for Health, Welfare, and Family Affairs, and covers the majority of the Korean population (approximately 97%).^15^ All medical institutions submit health care utilization–related data to ensure reimbursement, which are stored in the NHIS database. Established in January 2002, the NHIS database contains information from all hospitals, including admission and outpatient records (defined using the *International Statistical Classification of Diseases and Related Health Problems, Tenth Revision [ICD-10]*). The NHIS includes an eligibility database (according to age, sex, socioeconomic variables, and household income level), a medical treatment claims database (according to medical bills claimed by medical service practitioners for their medical expenses), a health examination database (results of general health examinations, questionnaires on lifestyle and behavior, and regular health check-ups for cardiovascular diseases and malignant neoplasms such as gastric, hepatic, colon, breast, and cervical cancer), and a medical care institution database (types of medical care institutions, location, and number of physicians). This customized research database is available to medical researchers for policy development and academic research. The data used in this study comprised all the NHIS claims records from 2002 to 2019.

### Ethical Considerations

Personal information in the Korean NHIS database was anonymized. Study protocols were approved by the official review committee of the Korean Government and the institutional review board of Korea University Anam Hospital and followed the Strengthening the Reporting of Observational Studies in Epidemiology (STROBE) reporting guideline reporting guidelines for cohort studies. The requirement for informed consent was waived by the institutional review board because the data used were deidentified.

### Study Participants

This study included women with breast cancer as the primary diagnosis on 2 or more occasions from January 1, 2003, to December 31, 2018. The women were aged 20 to 50 years when the first diagnosis was made. Breast cancer was defined by 1 of the following *ICD-10* diagnostic codes as the primary diagnosis: C50.X (malignant neoplasm of the breast), D05.X (carcinoma in situ of the breast), or D48.6 (neoplasm of uncertain or unknown behavior, breast). The first date of diagnosis registration in the NHIS database was assumed to be the date of diagnosis. A 1-year washout period was used in this study, meaning that women with the first claim record from January 1, 2002, to December 31, 2002 (which might have included those with a diagnosis before 2002) and those with the first claim records from January 1, 2019, to December 31, 2019, and who had a follow-up period of less than 1 year, were not included. The exclusion criteria were as follows: (1) patients who reported being menopausal at the time of breast cancer diagnosis, (2) patients with any of the diagnostic codes related to postmenopausal status (N95.0, N95.1, N95.2, N95.3, N95.8, N95.9, or E28.3), (3) patients who had been treated with aromatase inhibitors (letrozole, anastrozole, exemestane) or toremifene as an adjuvant hormone therapy, (4) patients who died after the diagnosis of breast cancer and before the prescription of tamoxifen, (5) patients who had diagnoses of the following uterine diseases before breast cancer was diagnosed (endometrial polyps [N84.0], endometrial hyperplasia [N85.0, N85.1], endometrial cancer and carcinoma in situ of endometrium [C54.1, D07.0], or other uterine malignant neoplasms [C54.0, C54.2, C54.3, C54.8, C54.9, and C55]), (6) patients who had the aforementioned diagnosis of uterine diseases that occurred between the diagnosis of breast cancer and the first treatment of tamoxifen, (7) patients whose prescriptions were switched between tamoxifen and aromatase inhibitors, and (8) patients with no health checkup data. All participants who satisfied all the inclusion and exclusion criteria were included in the study (eFigure in the Supplement). The participants were divided into 2 groups: the tamoxifen group (women who received tamoxifen only as the adjuvant hormone treatment for breast cancer) and the control group (women who did not receive adjuvant hormone treatment). For the subgroup analysis, the tamoxifen group was divided into 2 groups according to the duration of tamoxifen use: those using tamoxifen for 5 years or less and those using tamoxifen for more than 5 years.

### Study End Point and Other Variables

Medical histories of the participants were evaluated using *ICD-10* codes and the results of the questionnaire obtained during their health checkup visits. End points of this study were as follows: occurrence of endometrial polyps (N84.0), endometrial hyperplasia (N85.0, N85.1), endometrial cancer (C54.1, D07.0), other uterine malignant neoplasms (C54.0, C54.2, C54.3, C54.8, C54.9, and C55), and the combined results (N84.0, N85.0, N85.1, C54.1, D07.0, C54.0, C54.2, C54.3, C54.8, C54.9, and C55). Other uterine malignant neoplasms were defined as malignant neoplasms of the uterus that did not originate from the endometrium, as follows: C54.0, malignant neoplasm of the isthmus of the uterus; C54.2, malignant neoplasm of the myometrium; C54.3, malignant neoplasm of the fundus of the uterus; C54.8, malignant neoplasm of the overlapping sites of corpus of the uterus; C54.9, malignant neoplasm of the corpus of the uterus; and C55, malignant neoplasm of the uterus, part unspecified. The following variables were compared between the 2 groups: age, body mass index (BMI), waist circumference, history of diabetes, hypertension, dyslipidemia, polycystic ovary syndrome (PCOS), household income level, physical exercise level, current smoking status, alcohol consumption, blood pressure, serum fasting glucose, total cholesterol, high-density lipoprotein cholesterol, low-density lipoprotein cholesterol, and triglyceride levels. Physical exercise level was categorized into 3 groups (none, 1-4 times per week, and 5 or more times per week) according to the frequency of activity lasting at least 20 minutes per day.

### Statistical Analysis

The baseline demographic and clinical characteristics of the participants are summarized as mean (SD) for continuous variables and as numbers (percentages) for categorical variables. The *t* test and χ^2^ test were performed to compare the baseline characteristics of the groups, depending on the presence or absence of tamoxifen treatment. The incidence per 1000 person-years of each of the aforementioned uterine diseases was compared between the 2 groups using the Poisson regression model. Cox proportional hazard regression models were used to compare the risk of each disease outcome between the 2 groups. Multivariable Cox proportional hazard regression models were used to calculate hazard ratios (HRs) and 95% CIs after adjusting for age, BMI, history of diabetes , hypertension, dyslipidemia, polycystic ovary syndrome, gonadotropin-releasing hormone (GnRH) agonist treatment, and trastuzumab treatment. For the subgroup analysis, the cumulative duration of tamoxifen use was used as a time-dependent covariate. All statistical analyses were performed using SAS statistical software version 9.4 (SAS Institute Inc). All *P *values provided were 2-sided, and *P* < .05 was considered significant. Data were analyzed from April to December 2021.

## Results

Of the 78 320 premenopausal women with breast cancer (mean [SD] age, 42.1 [6.1] years), 34 637 (44.2%) were categorized as the tamoxifen group, and 43 683 (55.8%) as the control group. Compared with the women in the control group, those in the tamoxifen group had higher mean age, BMI, waist circumference, blood pressure, and fasting glucose levels; had poorer lipid profiles; were more likely to have a history of hypertension, diabetes, and dyslipidemia; had current smoking status; had treatment with GnRH agonist, trastuzumab, and metformin; had generally higher household income; and were less likely to have a history of PCOS (Table 1). The total number of uterine diseases that occurred during the study period were as follows in the tamoxifen group: 2882 endometrial polyps, 1911 endometrial hyperplasia, 307 endometrial cancer, and 71 other uterine cancers. In the control group the number of uterine diseases that occurred during the study period were as follows: 1426 endometrial polyps, 493 endometrial hyperplasia, 119 endometrial cancer, and 32 other uterine cancers. The comparison of the incidence per 1000 person-years of endometrial polyps, hyperplasia, cancer, other uterine cancers, and of the combined results is presented in Table 2. The incidence per 1000 person-years of newly diagnosed endometrial hyperplasia and endometrial cancer was approximately 6.6 times and 4.5 times higher in the tamoxifen group than in the control group, respectively. During the mean (SD) follow-up duration of 6.13 (4.15) years, the incidence of newly diagnosed endometrial polyps was 20.13 cases per 1000 person-years, that of endometrial hyperplasia was 13.49 cases per 1000 person-years, that of endometrial cancer was 2.01 cases per 1000 person-years, and that of other uterine cancers was 0.45 cases per 1000 person-years in tamoxifen users. The risk of endometrial cancer was higher in the tamoxifen group than in the control group (hazard ratio [HR], 3.77; 95% CI, 3.04-4.66) after adjusting for age, body mass index, history of diabetes, hypertension, dyslipidemia, polycystic ovary syndrome, GnRH agonist treatment, and trastuzumab treatment.

**Table 1.** Comparison of Baseline Characteristics According to Tamoxifen Use Among Premenopausal Women With Breast Cancer Diagnoses Between 2003 and 2018 in the Korean National Health Insurance Service Data

Variables	Patients, No. (%)		*P* value
	Tamoxifen (n = 34 637)	Control (n = 43 683)	
Age, mean (SD), y	43.9 (4.7)	40.8 (6.9)	<.001^a^
Body mass index, mean (SD)^b^	22.6 (3.3)	22.4 (6.3)	<.001^a^
Waist circumference, mean (SD), cm	74.1 (8.2)	73.6 (10.8)	<.001^a^
Medical history			
Diabetes	3139 (9.06)	3627 (8.30)	<.001^c^
Hypertension	3801 (10.97)	4272 (9.78)	<.001^c^
Dyslipidemia	11 001 (31.76)	12 331 (28.23)	<.001^c^
Polycystic ovary syndrome	439 (1.27)	672 (1.54)	.002^c^
Household income levels			
Low	7948 (22.95)	14 204 (32.52)	<.001^c^
Middle	17 270 (49.86)	19 963 (45.70)	
High	9419 (27.19)	9516 (21.78)	
Physical exercise level			
None	9508 (25.92)	12 415 (26.82)	<.001^c^
1-4 Times/week	11 653 (34.35)	16 104 (37.69)	
≥5 Times/week	13 476 (39.73)	15 164 (35.49)	
Current smoking	2423 (7.0)	2948 (6.75)	<.001^c^
Alcohol consumption (≥2 times per week)	1565 (4.52)	2067 (4.73)	.16^c^
Blood pressure, mean (SD), mm Hg			
Systolic	115.6 (13.8)	114.8 (13.7)	<.001^a^
Diastolic	72.6 (9.8)	72.1 (9.6)	<.001^a^
Fasting glucose, mean (SD), mg/dL	93.3 (15.8)	92.2 (17.3)	<.001^a^
Total cholesterol, mean (SD), mg/dL	188.3 (33.8)	188.1 (33.6)	.45^a^
High-density lipoprotein cholesterol, mean (SD), mg/dL	61.3 (21.2)	62.1 (21.2)	<.001^a^
Low-density lipoprotein cholesterol, mean (SD), mg/dL	108.4 (68.4)	110.7 (99.5)	.002^a^
Triglycerides, mean (SD), mg/dL	100.2 (71.9)	93.8 (67.0)	<.001^a^
Treated with gonadotropin-releasing hormone agonist	9211 (26.59)	527 (1.21)	<.001^c^
Treated with trastuzumab	3176 (9.17)	2271 (5.20)	<.001^c^
Treated with metformin	1584 (4.57)	1717 (3.93)	<.001^c^

SI conversion factors: To convert glucose to nanomoles per liter, multiply by 0.0555; high-density lipoprotein cholesterol to millimoles per liter, multiply by 0.259; low-density lipoprotein cholesterol to millimoles per liter, multiply by 0.259; total cholesterol to millimoles per liter, multiply by 0.259; triglycerides to millimoles per liter, multiply by 0.113.

^a^ Calculated with the *t* test.

^b^ Body mass index is calculated as weight in kilograms divided by height in meters squared.

^c^ Calculated with the χ^2^ test.

**Table 2.** Comparison of the Incidence of Uterine Diseases According to Tamoxifen Use Among Premenopausal Women With Breast Cancer in the Korean National Health Insurance Service Data From 2003 to 2018

Parameter	Incidence, cases per 1000 person-years (95% CI)		*P* value^a^
	Tamoxifen (n = 34 637)	Control (n = 43 683)	
Follow-up, median (IQR), y	3.63 (1.86-6.53)	5.40 (2.49-9.79)	NA
Endometrial polyp	20.13 (19.57-20.83)	5.5 (5.24-5.77)	<.001
Endometrial hyperplasia	13.49 (12.94-14.06)	2.06 (1.91-2.23)	<.001
Endometrial cancer	2.01 (1.94-2.18)	0.45 (0.38-0.53)	<.001
Other uterine cancers	0.45 (0.41-0.57)	0.16 (0.12-0.21)	<.001
Combination of results^b^	32.07 (31.81-32.24)	7.43 (7.13-7.74)	<.001

Abbreviation: NA, not applicable.

^a^ *P* values were calculated using Poisson regression analysis.

^b^ The combination of results included endometrial polyps, hyperplasia, cancer, and other uterine cancers.

Cox proportional hazard regression models were applied to compare the risk of each disease outcome and all composites between the 2 groups. The unadjusted HRs for endometrial hyperplasia and endometrial cancer were 5.55 (95% CI, 5.07-6.06) and 3.725 (95% CI, 3.03-4.57), respectively. The results of multivariable Cox regression analysis are shown in Table 3. After adjusting for several confounding factors, tamoxifen use was independently associated with the risk of endometrial polyps (HR, 3.90; 95% CI 3.65-4.16), hyperplasia (HR, 5.56; 95% CI 5.06-6.12), cancer (HR, 3.77; 95% CI 3.04-4.66), other uterine cancers (HR, 2.27; 95% CI 1.54-3.33), and the combined results (HR, 4.20; 95% CI 3.98-4.44). The Kaplan-Meier survival curves for the outcomes in the 2 groups are presented in the Figure.

**Table 3.** Risk of Endometrial Polyp, Endometrial Hyperplasia, Endometrial Cancer, Other Uterine Cancers, and Combined Results in Premenopausal Women With Breast Cancer in the Korean National Health Insurance Service Data^a^

Variable	HR (95% CI)				
	Endometrial polyp	Endometrial hyperplasia	Endometrial cancer	Other uterine cancers	Combined results
Tamoxifen use					
No	1 [Reference]	1 [Reference]	1 [Reference]	1 [Reference]	1 [Reference]
Yes	3.90 (3.65-4.16)	5.56 (5.06-6.12)	3.77 (3.04-4.66)	2.27 (1.54-3.33)	4.20 (3.98-4.44)
Age	0.97 (0.97-0.97)	1.0 (0.99-1.01)	1.01 (0.99-1.02)	1.05 (1.01-1.08)	0.98 (0.97-0.98)
Body mass index	0.98 (0.97-0.99)	1.01 (1.0-1.03)	1.04 (1.01-1.07)	1.0 (0.94-1.06)	0.99 (0.99-1.0)
Diabetes					
No	1 [Reference]	1 [Reference]	1 [Reference]	1 [Reference]	1 [Reference]
Yes	0.88 (0.78-0.98)	1.02 (0.90-1.17)	1.21 (0.90-1.62)	1.34 (0.76-2.38)	0.95 (0.87-1.04)
Hypertension					
No	1 [Reference]	1 [Reference]	1 [Reference]	1 [Reference]	1 [Reference]
Yes	1.05 (0.95-1.16)	1.0 (0.89-1.13)	0.90 (0.67-1.20)	0.68 (0.36-1.27)	1.04 (0.97-1.13)
Dyslipidemia					
No	1 [Reference]	1 [Reference]	1 [Reference]	1 [Reference]	1 [Reference]
Yes	1.36 (1.28-1.45)	1.33 (1.22-1.45)	1.65 (1.35-2.01)	1.55 (1.05-2.29)	1.33 (1.26-1.40)
Polycystic ovary syndrome					
No	1 [Reference]	1 [Reference]	1 [Reference]	1 [Reference]	1 [Reference]
Yes	1.34 (1.06-1.69)	1.45 (1.07-1.97)	2.11 (1.12-3.96)	2.79 (0.88-8.88)	1.31 (1.08-1.60)
Gonadotropin-releasing hormone agonist treatment					
No	1 [Reference]	1 [Reference]	1 [Reference]	1 [Reference]	1 [Reference]
Yes	0.95 (0.88-1.03)	1.26 (1.16-1.38)	1.23 (0.98-1.54)	0.91 (0.54-1.53)	1.06 (1.0-1.13)
Trastuzumab treatment					
No	1 [Reference]	1 [Reference]	1 [Reference]	1 [Reference]	1 [Reference]
Yes	0.65 (0.57-0.75)	1.12 (0.98-1.28)	1.21 (0.88-1.67)	1.84 (1.07-3.17)	0.85 (0.77-0.94)

Abbreviation: HR, hazard ratio.

^a^ Statistical analysis was performed using multivariable Cox proportional hazards regression analysis.

**Figure.**
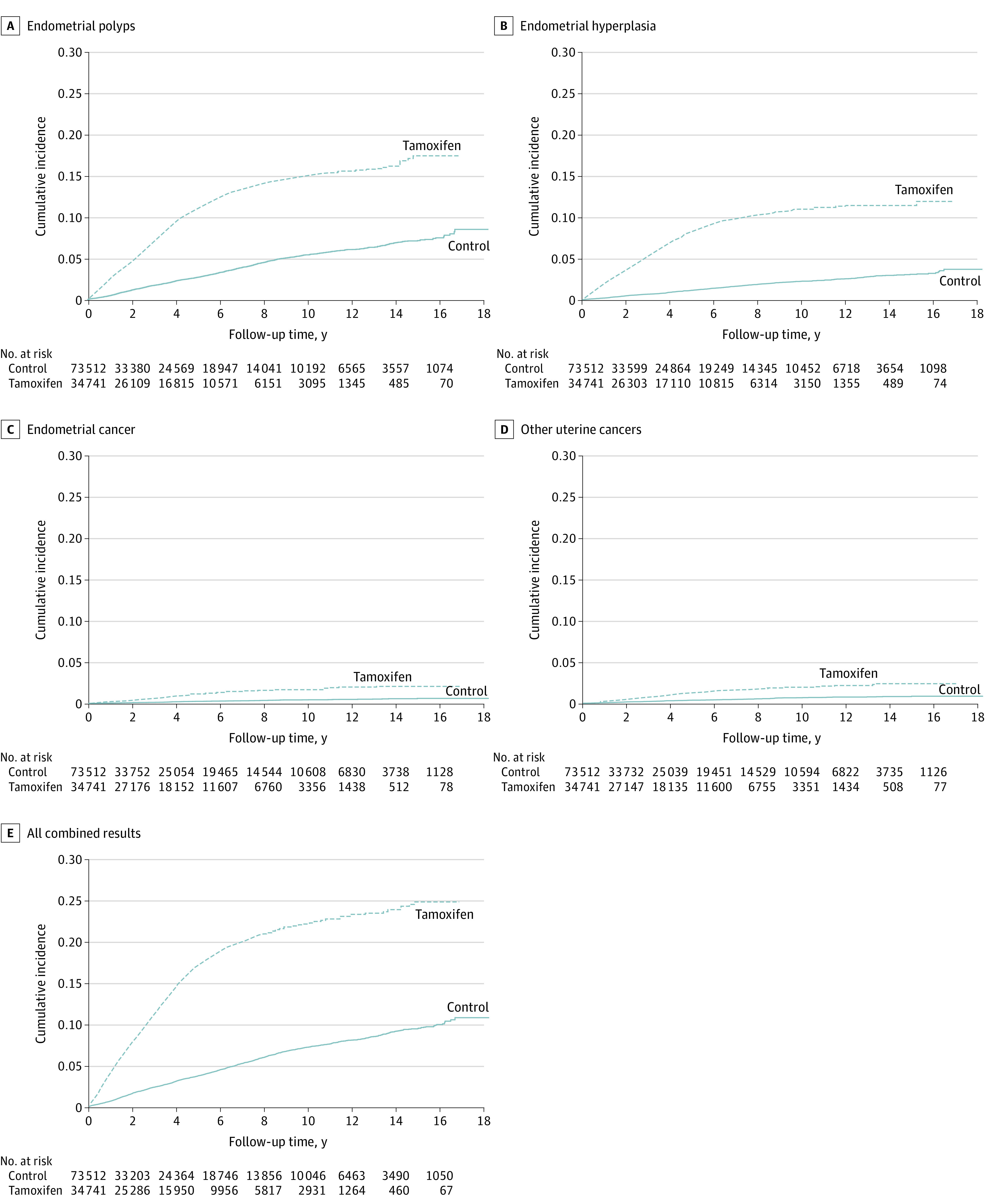
Cumulative Incidence of Uterine Diseases Among Premenopausal Women With Breast Cancer Treated With Tamoxifen or No Adjuvant Hormone Therapy

Among the women in the tamoxifen group, 9378 (27.1%) had prolonged tamoxifen treatment beyond 5 years. The results of the multivariable Cox regression analyses for the risk of each uterine disease comparing tamoxifen treatment for the group treated 5 or less and the group treated more than 5 years with the control group are presented in Table 4. After adjusting for the same variables used in the regression models, the HRs for each uterine disease were comparable between the 2 groups.

**Table 4.** Risk of Endometrial Polyp, Endometrial Hyperplasia, Endometrial Cancer, Other Uterine Cancers, and Combined Results According to the Duration of Tamoxifen Treatment in Premenopausal Women With Breast Cancer in the Korean National Health Insurance Service Data

Tamoxifen use	HR (95% CI)^a^				
	Endometrial polyp	Endometrial hyperplasia	Endometrial cancer	Other uterine cancers	Combined results
None	1 [Reference]	1 [Reference]	1 [Reference]	1 [Reference]	1 [Reference]
≤5 y	3.35 (3.10-3.62)	4.78 (4.27-5.35)	2.78 (2.17-3.56)	1.49 (0.95-2.31)	3.48 (3.26-3.71)
>5 y	2.36 (2.11-2.64)	3.43 (2.95-3.98)	2.90 (2.09-4.03)	1.79 (0.95-3.37)	2.32 (2.11-2.56)

Abbreviation: HR, hazard ratio.

^a^ Statistical analysis was performed using multivariable Cox proportional hazards regression analysis using the cumulative duration of tamoxifen use as a time-dependent covariate. The results were adjusted for age, body mass index, history of diabetes, history of hypertension, history of dyslipidemia, history of polycystic ovary syndrome, gonadotropin-releasing hormone agonist treatment, and trastuzumab treatment.

## Discussion

We conducted a population-based, retrospective, longitudinal cohort study using a nationwide cohort data with long-term follow-up and found that premenopausal women with breast cancer who received tamoxifen as an adjuvant hormone therapy had significantly increased risks of endometrial hyperplasia, polyps, carcinoma, and other uterine cancers compared with those who were not treated with adjuvant hormone therapy. Tamoxifen use was associated with an approximately 4-fold higher risk of developing endometrial cancer, even after controlling for several confounding factors, including age, BMI, diabetes, hypertension, dyslipidemia, PCOS, and GnRH agonist treatment.

In this study, the incidence of endometrial cancer was 2.01 cases per 1000 person-years in premenopausal women treated with tamoxifen and was comparable to that of tamoxifen-treated postmenopausal women with breast cancer (1.83/1000 person-years) observed in a previous multiethnic and multicenter cohort study in the US,^16^ and the incidence was even higher than that in various age groups (0.26-1.38 cases per 1000 person-years) observed in another population-based cohort study in South Korea.^17^ A meta-analysis conducted 10 years earlier by the Early Breast Cancer Trialists’ Collaborative Group included 20 trials (21 457 participants) and reported that 5 years of tamoxifen use was associated with increased incidence of endometrial cancer only in women older than 55 years.^18^ Another systematic review conducted around the same time also found that only women older than 50 years who received tamoxifen, and not younger patients, had a higher risk of endometrial cancer than those who did not use tamoxifen.^19^ Unlike previous Western studies, recent studies conducted in East Asian populations showed contrasting results. A population-based cohort study^20^ with a 14-year study period included 39 216 women with nonmetastatic breast cancer in Taiwan. The study found that both older (aged ≥50 years) and younger (aged 40-49 years) patients treated with tamoxifen had a higher risk (HR, 3.74; 95% CI, 1.65-8.48) of endometrial cancer than those who did not use tamoxifen.^20^ A recent retrospective cohort study^17^ using Health Insurance Review and Assessment Service claims data from South Korea included 60 545 women who had new diagnoses of breast cancer between 2010 and 2015. The study found that the use of tamoxifen was associated with an increased risk of endometrial cancer among women aged 40 to 49 years (HR, 2.12; 95% CI, 1.07-4.21).^17^ However, these studies could not assess the menopausal status of women and did not control for some confounding factors in their analyses, such as BMI, although differences in baseline characteristics were observed between the tamoxifen and control groups.^19,20^

Tamoxifen is also associated with endometrial hyperplasia and polyp formation.^12^ Although these benign diseases are not life-threatening, they might be the most common endometrial pathologies occurring in tamoxifen users. These pathologies lead to additional medical costs or invasive procedures.^11^ The present study showed a greater risk of benign diseases in premenopausal tamoxifen users than those reported in previous clinical studies.^21^ Furthermore, we reaffirmed the increased risk of uterine cancers other than endometrial cancer such as sarcoma in premenopausal tamoxifen users.^12^ Tumor-promoting effects of tamoxifen on the uterus might be the mechanism that underlies those findings^4^; however, it is also possible that women who were treated with tamoxifen visited gynecologic clinics more frequently than women who did not, resulting in a higher detection rate of asymptomatic lesions.

The American College of Obstetricians and Gynecologists Committee advised that premenopausal women treated with tamoxifen have no known increased risk of uterine cancer and did not recommend any additional monitoring beyond routine gynecologic care.^12^ These recommendations were based on previous studies^18,19,22,23^ that reported no associations between endometrial cancer and tamoxifen use among premenopausal women. However, most of these studies were outdated, with a small number of premenopausal women. These were hospital-based studies and conducted mostly in Western populations. Our findings clearly indicate that clinicians should consider the risks of endometrial cancer and other uterine malignant neoplasms among tamoxifen users, regardless of menopausal status. Furthermore, the median age at which East Asian women receive a breast cancer diagnosis is approximately 10 years lower than that of Western women.^14,24^ This makes it particularly important for clinicians to be aware of the risks of various uterine diseases in young premenopausal breast cancer survivors receiving adjuvant hormone therapies.

Several expected factors associated with risk for endometrial cancer were reconfirmed in this study, including BMI, history of dyslipidemia, and PCOS; however, diabetes and hypertension were not confirmed.^25,26^ GnRH agonist treatment is associated with a higher risk of endometrial hyperplasia, although it has been shown that this treatment and the subsequently induced hypoestrogenism reduce the risk of endometrial diseases.^27^ This could be a consequence of the higher incidence of GnRH agonist use in the tamoxifen group. However, its suppressive effect on progesterone levels in premenopausal women, which has a protective role in the endometrium, should be considered.^28^ Interestingly, a history of trastuzumab treatment increased the risk of uterine cancers other than endometrial cancer, and the risk of endometrial polyps was reduced by approximately 50%. These findings should be investigated in future studies.

### Limitations

Our study had limitations. First, considering that asymptomatic patients might be less likely to visit a gynecologic clinic, the incidence of uterine diseases might have been underestimated in the NHIS database. Furthermore, annual gynecologic screening is recommended in Korea for patients with breast cancer during the tamoxifen-treatment period and would help in diagnosing uterine diseases in patients more easily than in those who are not treated with tamoxifen. However, this limitation might be compensated by the high accessibility of health service in South Korea. Second, there was no information on clinical symptoms, histological findings, tumor stage, or tumor grade in the data. Therefore, we could not perform analyses according to the different types of breast cancer. Further studies are required to investigate whether these variables affect the association between tamoxifen and gynecologic diseases. Third, there were no data on whether the breast cancer in the patients was estrogen receptor–positive or estrogen receptor–negative. Although women with estrogen receptor–positive breast cancer who were not treated with tamoxifen would be the most appropriate control group for the tamoxifen group, such enrollment might be difficult considering the inherent limitation of using a claim-based database. Fourth, genetic variation data related to breast and endometrial cancers, such as *BRCA* and *MMR* genes, were not available; further studies are needed to confirm the effect of genetic status on the association between tamoxifen use and the risk of uterine diseases. Fifth, owing to the retrospective nature of the study, we cannot exclude the possibility that the patients enrolled in the tamoxifen group had an undiagnosed uterine disease at the time of tamoxifen exposure. Diagnostic hysteroscopy immediately before beginning tamoxifen therapy in future prospective studies could overcome this limitation.

## Conclusions

In this longitudinal cohort study, premenopausal Korean women with breast cancer who received tamoxifen as adjuvant hormone therapy had a significantly higher risk of endometrial hyperplasia, polyps, carcinoma, and other uterine cancers than those not treated with tamoxifen. Clinicians managing premenopausal tamoxifen users should consider the risks of these uterine diseases and counsel patients accordingly.

## Notes

Supplement. **eFigure.** Flowchart of This Study

## References

[1] Bray F, Ferlay J, Soerjomataram I, Siegel RL, Torre LA, Jemal A. Global cancer statistics 2018: GLOBOCAN estimates of incidence and mortality worldwide for 36 cancers in 185 countries. CA Cancer J Clin. 2018;68(6):394-424. doi:10.3322/caac.21492 30207593

[2] Goldstein SR. Drugs for the gynecologist to prescribe in the prevention of breast cancer: current status and future trends. Am J Obstet Gynecol. 2000;182(5):1121-1126. doi:10.1067/mob.2000.105941 10819845

[3] National Comprehensive Cancer Network. Breast cancer. Accessed April 20, 2022. https://www.nccn.org/guidelines/guidelines-detail?category=1&id=1419

[4] Osborne CK. Tamoxifen in the treatment of breast cancer. N Engl J Med. 1998;339(22):1609-1618. doi:10.1056/NEJM199811263392207 9828250

[5] No authors listed. Tamoxifen for early breast cancer: an overview of the randomised trials. Early Breast Cancer Trialists’ Collaborative Group. Lancet. 1998;351(9114):1451-1467. doi:10.1016/S0140-6736(97)11423-4 9605801

[6] Rutqvist LE, Johansson H; Stockholm Breast Cancer Study Group. Long-term follow-up of the randomized Stockholm trial on adjuvant tamoxifen among postmenopausal patients with early stage breast cancer. Acta Oncol. 2007;46(2):133-145. doi:10.1080/02841860601034834 17453361

[7] Swerdlow AJ, Jones ME; British Tamoxifen Second Cancer Study Group. Tamoxifen treatment for breast cancer and risk of endometrial cancer: a case-control study. J Natl Cancer Inst. 2005;97(5):375-384. doi:10.1093/jnci/dji057 15741574

[8] d’Arailh AS, Michy T, Pioud R, Dravet F, Classe JM. Uterine abnormalities in non menopausal women who received tamoxifen for breast cancer adjuvant therapy [in French]. Gynecol Obstet Fertil. 2007;35(12):1215-1219. doi:10.1016/j.gyobfe.2007.10.006 18035581

[9] Kim HS, Jeon YT, Kim YB. The effect of adjuvant hormonal therapy on the endometrium and ovary of breast cancer patients. J Gynecol Oncol. 2008;19(4):256-260. doi:10.3802/jgo.2008.19.4.256 19471651 PMC2676481

[10] Gu R, Jia W, Zeng Y, . A comparison of survival outcomes and side effects of toremifene or tamoxifen therapy in premenopausal estrogen and progesterone receptor positive breast cancer patients: a retrospective cohort study. BMC Cancer. 2012;12:161. doi:10.1186/1471-2407-12-161 22548922 PMC3503787

[11] Jeon SJ, Lee JI, Lee M, . Endometrial polyp surveillance in premenopausal breast cancer patients using tamoxifen. Obstet Gynecol Sci. 2017;60(1):26-31. doi:10.5468/ogs.2017.60.1.26 28217668 PMC5313360

[12] Committee on Gynecologic Practice. Committee opinion no. 601: tamoxifen and uterine cancer. Obstet Gynecol. 2014;123(6):1394-1397. doi:10.1097/01.AOG.0000450757.18294.cf 24848920

[13] American Cancer Society. Hormone therapy for breast cancer. Accessed April 20, 2022. https://www.cancer.org/cancer/breast-cancer/treatment/hormone-therapy-for-breast-cancer.html

[14] Kang SY, Kim YS, Kim Z, ; Korean Breast Cancer Society. Breast cancer statistics in Korea in 2017: data from a breast cancer registry. J Breast Cancer. 2020;23(2):115-128. doi:10.4048/jbc.2020.23.e24 32395372 PMC7192743

[15] Cheol Seong S, Kim YY, Khang YH, . Data resource profile: the national health information database of the National Health Insurance Service in South Korea. Int J Epidemiol. 2017;46(3):799-800. doi:10.1093/ije/dyw253 27794523 PMC5837262

[16] Chlebowski RT, Schottinger JE, Shi J, Chung J, Haque R. Aromatase inhibitors, tamoxifen, and endometrial cancer in breast cancer survivors. Cancer. 2015;121(13):2147-2155. doi:10.1002/cncr.29332 25757699 PMC4565775

[17] Choi S, Lee YJ, Jeong JH, . Risk of endometrial cancer and frequencies of invasive endometrial procedures in young breast cancer survivors treated with tamoxifen: a nationwide study. Front Oncol. 2021;11:636378. doi:10.3389/fonc.2021.636378 34150613 PMC8209428

[18] Davies C, Godwin J, Gray R, ; Early Breast Cancer Trialists’ Collaborative Group (EBCTCG). Relevance of breast cancer hormone receptors and other factors to the efficacy of adjuvant tamoxifen: patient-level meta-analysis of randomised trials. Lancet. 2011;378(9793):771-784. doi:10.1016/S0140-6736(11)60993-8 21802721 PMC3163848

[19] Iqbal J, Ginsburg OM, Wijeratne TD, . Endometrial cancer and venous thromboembolism in women under age 50 who take tamoxifen for prevention of breast cancer: a systematic review. Cancer Treat Rev. 2012;38(4):318-328. doi:10.1016/j.ctrv.2011.06.009 21775065

[20] Chu SC, Hsieh CJ, Wang TF, Hong MK, Chu TY. Younger tamoxifen-treated breast cancer patients also had higher risk of endometrial cancer and the risk could be reduced by sequenced aromatase inhibitor use: a population-based study in Taiwan. Ci Ji Yi Xue Za Zhi. 2019;32(2):175-180. doi:10.4103/tcmj.tcmj_17_19 32269951 PMC7137368

[21] Chalas E, Costantino JP, Wickerham DL, . Benign gynecologic conditions among participants in the Breast Cancer Prevention Trial. Am J Obstet Gynecol. 2005;192(4):1230-1237. doi:10.1016/j.ajog.2004.12.083 15846210

[22] Fisher B, Costantino JP, Wickerham DL, . Tamoxifen for prevention of breast cancer: report of the National Surgical Adjuvant Breast and Bowel Project P-1 Study. J Natl Cancer Inst. 1998;90(18):1371-1388. doi:10.1093/jnci/90.18.1371 9747868

[23] Fisher B, Costantino JP, Redmond CK, Fisher ER, Wickerham DL, Cronin WM. Endometrial cancer in tamoxifen-treated breast cancer patients: findings from the National Surgical Adjuvant Breast and Bowel Project (NSABP) B-14. J Natl Cancer Inst. 1994;86(7):527-537. doi:10.1093/jnci/86.7.527 8133536

[24] Lin CH, Chuang PY, Chiang CJ, . Distinct clinicopathological features and prognosis of emerging young-female breast cancer in an East Asian country: a nationwide cancer registry-based study. Oncologist. 2014;19(6):583-591. doi:10.1634/theoncologist.2014-0047 24807917 PMC4041679

[25] Friedenreich CM, Biel RK, Lau DC, . Case-control study of the metabolic syndrome and metabolic risk factors for endometrial cancer. Cancer Epidemiol Biomarkers Prev. 2011;20(11):2384-2395. doi:10.1158/1055-9965.EPI-11-0715 21921255

[26] Ignatov A, Ortmann O. Endocrine risk factors of endometrial cancer: polycystic ovary syndrome, oral contraceptives, infertility, tamoxifen. Cancers (Basel). 2020;12(7):E1766. doi:10.3390/cancers12071766 32630728 PMC7408229

[27] Emons G, Gründker C. The role of gonadotropin-releasing hormone (GnRH) in endometrial cancer. Cells. 2021;10(2):292. doi:10.3390/cells10020292 33535622 PMC7912811

[28] Gompel A. Progesterone and endometrial cancer. Best Pract Res Clin Obstet Gynaecol. 2020;69:95-107. doi:10.1016/j.bpobgyn.2020.05.003 32732107

