# Supplementary material for: Risk of Endometrial Polyps, Hyperplasia, Carcinoma, and Uterine Cancer After Tamoxifen Treatment in Premenopausal Women With Breast Cancer
Source: JAMA Netw Open. 2022 Nov 28;5(11):e2243951. doi: 10.1001/jamanetworkopen.2022.43951 (PMC9706361; doi:10.1001/jamanetworkopen.2022.43951)
Supplement: Supplement. — eFigure. Flowchart of This Study [file jamanetwopen-e2243951-s001.pdf]

Supplemental Online Content

Ryu KJ, Kim MS, Lee JY, et al. Risk of endometrial polyps, hyperplasia, carcinoma, and uterine cancer after tamoxifen treatment in premenopausal women with breast cancer. *JAMA Netw Open*. 2022;5(11):e2243951. doi:10.1001/jamanetworkopen.2022.43951

eFigure. Flowchart of This Study

This supplemental material has been provided by the authors to give readers additional information about their work.

© 2022 Ryu KJ et al. *JAMA Network Open.*

Supplement Figure

eFigure. Flowchart of This Study

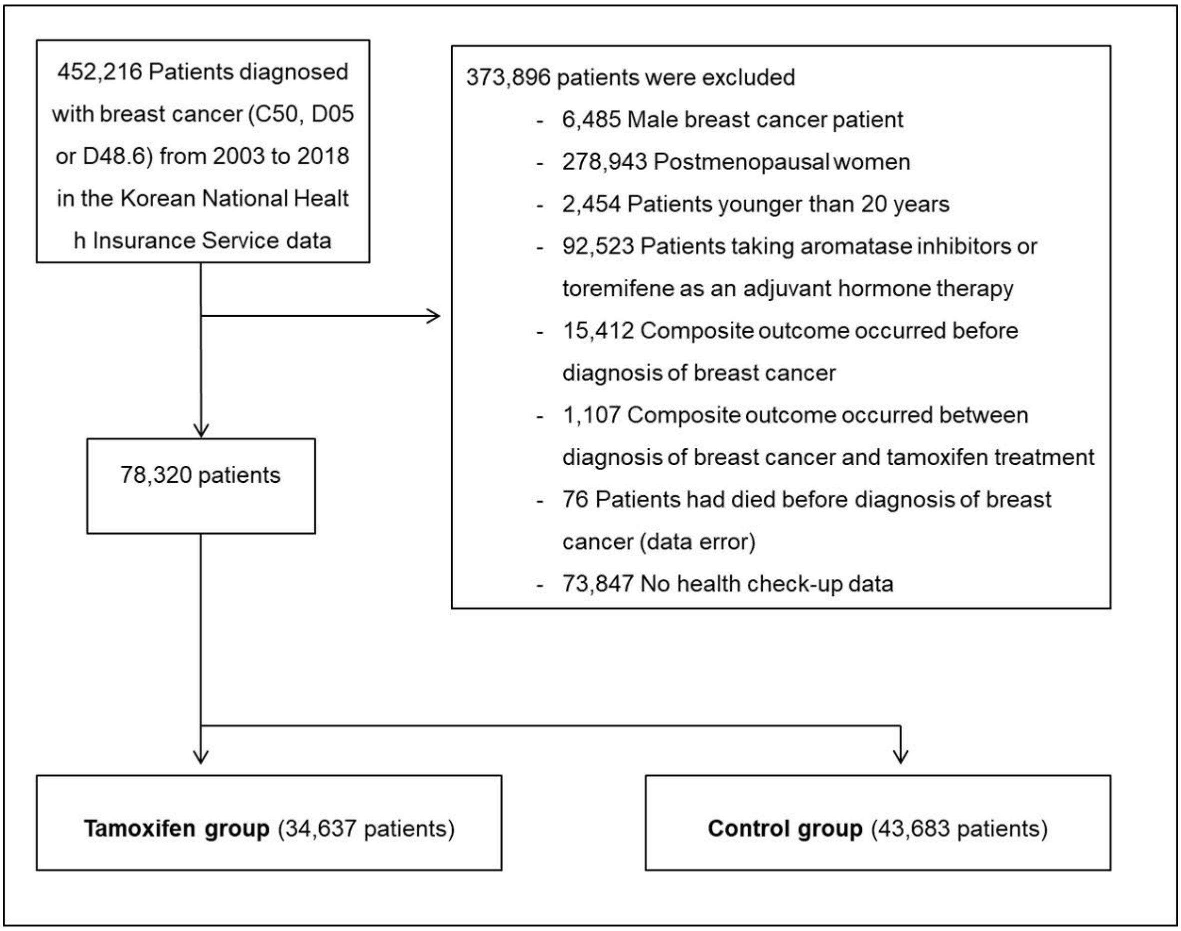

This population-based retrospective cohort study using data obtained from the Korean National Health Insurance Service included premenopausal women diagnosed with breast cancer from 2002 to 2019. The participants were divided into two groups: the tamoxifen group (women who received tamoxifen only as the adjuvant hormone treatment for breast cancer) and the control group (women who did not receive adjuvant hormone treatment).

© 2022 Ryu KJ et al. *JAMA Network Open.*
